# Factors associated with self-reported diabetes according to the 2013 National Health Survey

**DOI:** 10.1590/S1518-8787.2017051000011

**Published:** 2017-06-01

**Authors:** Deborah Carvalho Malta, Regina Tomie Ivata Bernal, Betine Pinto Moehlecke Iser, Célia Landmann Szwarcwald, Bruce Bartholow Duncan, Maria Inês Schmidt

**Affiliations:** I Departamento de Enfermagem Materno Infantil e Saúde Pública. Escola de Enfermagem. Universidade Federal de Minas Gerais. Belo Horizonte, MG, Brasil; II Núcleo de Pesquisas Epidemiológicas em Nutrição e Saúde. Universidade de São Paulo. São Paulo, SP, Brasil; III Programa de Pós-Graduação em Epidemiologia. Universidade Federal do Rio Grande do Sul. Porto Alegre, RS, Brasil; IV Faculdade de Medicina. Universidade do Sul de Santa Catarina. Tubarão, SC, Brasil; V Instituto de Comunicação e Informação Científica e Tecnológica em Saúde. Fundação Oswaldo Cruz. Rio de Janeiro, RJ, Brasil

**Keywords:** Adult, Diabetes Mellitus, epidemiology, Diagnostic Self Evaluation, Risk Factors, Socioeconomic Factors, Health Surveys, Adulto, Diabetes Mellitus, epidemiologia, Autoavaliação Diagnóstica, Fatores de Risco, Fatores Socioeconômicos, Inquéritos Epidemiológicos

## Abstract

**OBJECTIVES:**

To analyze the factors associated with self-reported diabetes among adult participants of the National Health Survey (PNS).

**METHODS:**

Cross-sectional study using data of the PNS carried out in 2013, from interviews with adults (≥ 18 years) of 64,348 Brazilian households. The prevalence of self-reported diabetes, assessed by the question “Has a doctor ever told you that you have diabetes?,” was related to sociodemographic characteristics, lifestyle, self-reported chronic disease, and self-evaluation of the health condition. Prevalence ratios were adjusted according to age, sex, and schooling by Poisson regression with robust variance.

**RESULTS:**

The diagnosis of diabetes was reported by 6.2% of respondents. Its crude prevalence was higher in women (7.0% vs. 5.4%), and among older adults, reaching 19.8% in the elderly. Black adults who received less schooling showed higher prevalence. Among those classified as obese, 11.8% reported having diabetes. Ex-smokers, those insufficiently active and those who consume alcohol abusively reported diabetes more often. Differences were not verified in eating habits among adults who reported, or did not, diabetes. A relation between diabetes and hypertension was found.

**CONCLUSIONS:**

After adjustment according to age, schooling and sex, diabetes was shown to be associated with higher age, lower schooling, past smoking, overweight and obesity, and hypertension, as well as with a self-declared poor state of health, indicating a pattern of risk factors common to many chronic non-communicable diseases and the association of the disease with morbidity.

## RESUMO

**OBJETIVOS:**

Analisar os fatores associados ao diabetes autorreferido entre adultos entrevistados na Pesquisa Nacional de Saúde (PNS).

**MÉTODOS:**

Estudo transversal utilizando dados da PNS realizada em 2013, a partir de entrevistas com adultos (≥ 18 anos) de 64.348 domicílios brasileiros. A prevalência de diabetes autorreferido, avaliada pela pergunta “Algum médico já lhe disse que o sr(a) tem diabetes?”, foi relacionada a características sociodemográficas, estilos de vida, doença crônica autorreferida e autoavaliação do estado de saúde. Razões de prevalência foram ajustadas segundo idade, sexo e escolaridade por regressão de Poisson com variância robusta.

**RESULTADOS:**

O diagnóstico de diabetes foi referido por 6,2% dos entrevistados. Sua prevalência bruta foi maior em mulheres (7,0 *vs.* 5,4%), e entre adultos de maior idade, chegando a 19,8% dos idosos. Os adultos de cor preta e de menor escolaridade apresentaram prevalências maiores. Entre os classificados como obesos, 11,8% referiram ter diabetes. Ex-fumantes, aqueles insuficientemente ativos e que consomem álcool abusivamente referiram diabetes mais frequentemente. Não foram verificadas diferenças nos hábitos alimentares entre adultos que referiram ou não diabetes. Verificou-se relação entre diabetes e a ocorrência de hipertensão arterial.

**CONCLUSÕES:**

Após ajustes por idade, escolaridade e sexo, o diabetes mostrou-se associado com maior idade, menor escolaridade, fumo no passado, sobrepeso e obesidade, e hipertensão arterial sistêmica, bem como com estado de saúde auto-declarado como ruim, indicando um padrão de fatores de risco comum a várias doenças crônicas não transmissíveis e associação da doença com morbidade.

## INTRODUCTION

The prevalence of diabetes is growing globally, partly due to the demographic transition, but also due to urbanization and to unhealthy lifestyles, such as sedentariness and inadequate eating habits, which results in metabolic changes and excess weight^1,a^. Studies show that interventions that lead to lifestyle changes, such as the practice of physical activity and adoption of a healthy eating pattern, can prevent diabetes mellitus^2-4^.

The World Health Organization (WHO) estimates that diabetes mellitus has been responsible for 1.5 million deaths in 2012^a^. Due to its numerous comorbidities and complications, diabetes affects the occupational and social life of those with the condition and causes direct and indirect costs to them and to society.

Estimates based on data from 193 countries indicate global expenditures on diabetes of 376 billion U.S. dollars in 2010, representing 12% of total health expenditures, and with projection to reach 490 billion dollars in 2030^5^. A study in Brazil, which evaluated data from 1,000 patients seen at different levels of health care in eight Brazilian cities, estimated a total cost of more than two thousand dollars per patient per year, 63% of which were related to direct costs^6^.

The National Health Survey (PNS), a major national representative survey, was conducted in Brazil in 2013^b^. In addition to diabetes and other chronic diseases, modules were included on demographic, nutritional factors, lifestyle, use of medicines, among others, which enables comprehensive analysis about the factors associated with self-reported diabetes.

This study aimed to analyze the factors associated with self-reported diabetes among adults interviewed on PNS. Specifically, sociodemographic factors, overweight, behavioral risk and protective factors, eating habits and smoking will be assessed, as well as perception of health and comorbidities, such as hypertension and dyslipidemia.

## METHODS

This is a cross-sectional study using data of the PNS carried out in 2013, household survey that employed sampling consisting of three-stage conglomerate, with stratification of the primary sampling units. The census tracts or set of tracts were the primary units, the households were the second-stage units, and the residents aged 18 years or older, third-stage units.

Within each household, one resident aged 18 years or more was selected from the list of residents prepared at the time of the interview to answer to a specific questionnaire^7^. The sample drafted consisted of 81,357 households, 69,994 of which, being occupied, were considered eligible for the research. At the end, interviews were held in 64,348 households (non-response rate of 8.1%)^8^.

The outcome analyzed in this study was the prevalence of self-reported diabetes, according to the positive answer to the question “Has a doctor ever told you that you have diabetes?” The explanatory variables were: a) sociodemographic characteristics: sex, age, schooling, race/color); b) habits and lifestyles considered risk factors (insufficiency in the four domains of physical activity [leisure, work, commute to work, and home], smoking, consumption of red meat with fat, report of high salt intake and abusive consumption of alcohol); c) habits and lifestyles considered protective factors: recommended intake of fruits and vegetables (5 or more servings per day); d) metabolic risk factors for cardiovascular disease (overweight and obesity, hypertension, high cholesterol); e) self-assessment of one’s state of health. Bivariate analysis and calculation of prevalence were performed with 95% confidence interval (95%CI). Prevalence ratios (PR) were calculated by the method of Poisson regression with robust variance, and presented both crude and adjusted according to age, sex and schooling for the global sample and by age and schooling in each category of sex. We used the version 12.1 Stata (Stata Corp, College Station, USA) for data processing and statistical analysis.

This study was approved by the *Comissão Nacional de Ética em Pesquisa* (CONEP – National Commission of Ethics in Research) in June 2013 (no. 328,159).

## RESULTS

Table 1 describes the prevalence of self-reported diabetes in the sample and separately for men and women, according to various factors often associated with the diagnosis of diabetes. The prevalence of self-reported diabetes was always greater in women and increased notably with age, reaching 19.8% in those with more than 65 years. The prevalence decreased as schooling increased, and tended to be higher in black people. Diabetes was mentioned more often in those who reported practicing very little physical activity. Ex-smokers reported diabetes more frequently than smokers and non-smokers.

**Table 1:** Prevalence of diabetes in adults by sex, according to sociodemographic factors. National Health Survey, Brazil, 2013.

Variable	Total		Male		Female	
		
	%	95%CI	%	95%CI	%	95%CI
Total	6.2	5.9–6.6	5.4	4.8–5.9	7	6.5–7.5
Age (years)						
18–24	0.5	0.3–0.8	0.4	0.1–0.7	0.6	0.2–1.1
25–34	0.8	0.6–1.1	0.8	0.4–1.2	0.9	0.6-1.2
35–44	3	2.4–3.5	2.5	1.7–3.3	3.3	2.6–4.1
45–54	6.5	5.8–7.3	5.7	4.6–6.8	7.3	6.2–8.4
55–64	13.5	12–15	12.1	9.7–14.4	14.8	12.9–16.7
≥ 65	19.8	18.2–21.4	18	15.2–20.7	21.2	19.1–23.4
Schooling (years)						
Illiterate/some elementary or middle school	9.6	8.9–10.3	6.7	5.8–7.6	12.3	11.3–13.4
Elementary or middle school/some high school	5.4	4.4–6.3	5.4	3.8–6.9	5.4	4.3–6.4
High school/some college	3.4	3–3.9	3.6	2.8–4.3	3.3	2.7–3.9
College degree	4.2	3.3–5	5.7	4–7.4	3.1	2.2–3.9
Race/color^a^						
White	6.7	6.1–7.2	6	5.2–6.8	7.3	6.5–8
Black	7.2	5.8–8.5	5.4	3.2–7.6	8.7	7.1–10.4
Pardo	5.5	5.1–6	4.6	3.9–5.2	6.4	5.8–7
Categories of BMI^b^						
Underweight/normal (< 25 kg/m^2^)	3.3	2.8–3.8	3.4	2.7–4.2	3.2	2.5–3.8
Overweight (between 25 and 29.9 kg/m^2^)	6.9	6.1–7.7	6.5	5.3–7.6	7.5	6.4–8.6
Obesity (≥ 30 kg/m^2^)	11.8	10.4–13.1	10.3	8.5–12.1	13	11.2–14.8
Insufficiently active in the 4 domains^c^						
No	4.9	4.5–5.3	4	3.5–4.5	5.9	5.2–6.6
Yes	7.8	7.3–8.4	7.4	6.4–8.5	8.1	7.4–8.7
Smoking						
Non-smoker	5.4	5–5.8	4.1	3.6–4.7	6.2	5.7–6.8
Ex-smoker	10.8	9.8–11.9	9.8	8.3–11.3	12.2	10.7–13.8
Smoker	4.8	3.9–5.7	4.2	2.9–5.5	5.7	4.5–6.8
Recommended consumption of fruits and vegetables (5 or more servings per day)						
Yes	8	7–8.9	7.8	6.4–9.2	8.1	6.8–9.3
No	5.9	5.5–6.3	5	4.4–5.6	6.8	6.3–7.3
Consumption of red meat with fat						
Yes	5.4	4.8–6	4.4	3.6–5.2	6.7	5.8–7.6
No	6.7	6.2–7.1	6	5.3–6.7	7.1	6.6–7.7
High salt intake						
No	6.6	6.2–7	5.7	5.1–6.3	7.3	6.8–7.8
Yes	4.2	3.4–5	3.5	2.4–4.5	5.1	3.8–6.3
Abusive consumption of alcohol						
No	2.6	1.8–3.3	2.4	1.6–3.3	3	1.2–4.7
Yes	6.5	6.1–6.8	5.7	5.1–6.3	7.1	6.6–7.6
Systemic hypertension						
No	3	2.7–3.2	2.8	2.4–3.2	3.1	2.8–3.5
Yes	18.3	17–19.5	16.9	14.7–19	19.2	17.6–20.8
High cholesterol						
No	4.5	4.1–4.8	4.1	3.6–4.7	4.8	4.4–5.2
Yes	18.5	16.9–20.1	16.7	13.9–19.4	19.6	17.6–21.5
Assessment of the state of health						
Good	2.6	2.3–2.8	2.4	2–2.8	2.7	2.4–3.1
Regular	12.2	11.3–13.2	12	10.4–13.6	12.4	11.2–13.5
Poor/very poor	19.1	16.9–21.2	14.2	11–17.4	22.1	19.3–25

BMI: body mass index.

^a^ The categories yellow and indigenous were excluded from these analyses.

^b^ 32% missing data for BMI.

^c^ Physical activity domains: leisure, work, commute to work, and home.

Regarding eating habits, the prevalence was higher among those who reported consuming five or more servings of fruits and vegetables (8.0% vs. 5.9%); the prevalence was lower among those who reported consuming red meat with fat and excess salt, in relation to the others. A greater frequency of self-reported diabetes was observed in individuals who reported drinking alcohol excessively (6.6% vs. 2.6% among the others).

Among those classified as obese, 11.8% reported having diabetes, in contrast to those of low weight or eutrophic, of which only 3.3% reported having diabetes. The frequency of self-reported diabetes was higher among those who reported previous diagnosis of hypertension or high cholesterol. In those who considered their health poor or very poor, the rate reached 19.1%.

Table 2 describes the magnitude of these associations, which varied considerably among the factors analyzed. For example, in individuals aged more than 65 years, the prevalence is 37 times higher than in youngest individuals; regarding schooling, individuals with higher schooling showed lower PR, reaching PR = 0.4 overall for those with a college degree and PR = 0.3 among women with college degree, compared with those illiterate or with some elementary or middle school. In general, known risk factors (tobacco, BMI, consumption of fruit and vegetables, meat with fat, alcohol, excess salt, physical inactivity, hypertension, cholesterol, health assessment) showed statistically significant associations with diabetes, except for a lack of predominance among black individuals in relation to the others. In men, the inverse association with the schooling was not statistically significant.

**Table 2:** Prevalence ratio (crude) and 95% confidence interval for diabetes in adults, stratified by sex, according to sociodemographic characteristics, clinical conditions and lifestyles. National Health Survey, Brazil, 2013.

Variable	Total		Male		Female	
		
	PR_crude_	95%CI	PR_crude_	95%CI	PR_crude_	95%CI
Age (years)						
18–24	1		1		1	
25–34	1.6	0.9–2.8	1.9	0.8–4.6	1.4	0.7–2.7
35–44	5.6	3.3–9.4	6.2	2.9–13.5	5.2	2.6–10.3
45–54	12.4	7.7–20.1	14	6.8–29	11.3	5.9–21.9
55–64	25.7	15.7–42	29.7	14.4–61.3	22.9	11.8–44.4
≥ 65	37.6	23.1–61.2	44.3	21.7–90.6	32.9	17–63.6
Schooling (years)						
Illiterate/some elementary or middle school	1		1		1	
Elementary or middle school/some high school	0.6	0.5–0.7	0.8	0.6–1.1	0.4	0.4–0.5
High school/some college	0.4	0.3–0.4	0.5	0.4–0.7	0.3	0.2–0.3
College degree	0.4	0.3–0.5	0.8	0.6–1.2	0.3	0.2–0.3
Race/color^a^						
White	1		1		1	
Black	1.1	0.9–1.3	0.9	0.6–1.4	1.2	1–1.5
Pardo	0.8	0.7–0.9	0.8	0.6–0.9	0.9	0.8–1
Categories of BMI^b^						
Underweight/normal (< 25 kg/m^2^)	1		1		1	
Overweight (between 25 and 29.9 kg/m^2^)	2.1	1.8–2.5	1.9	1.5–2.4	2.4	1.9–3
Obesity (≥ 30 kg/m^2^)	3.6	3–4.3	3	2.3–3.9	4.1	3.3–5.2
Insufficiently active in the 4 domains^c^						
No	1		1		1	
Yes	1.6	1.4–1.8	1.9	1.5–2.2	1.4	1.2–1.6
Smoking						
Non-smoker	1		1		1	
Ex-smoker	2	1.8–2.3	2.4	1.9–2,9	2	1.7–2.3
Smoker	0.9	0.7–1.1	1	0.7–1.4	0.9	0.7–1.1
Recommended consumption of fruits and vegetables (5 or more servings per day)						
Yes	1		1		1	
No	0.7	0.6–0.9	0.6	0.5–0.8	0.8	0.7–1
Consumption of red meat with fat						
Yes	1		1		1	
No	1.2	1.1–1.4	1.4	1.1–1.7	1.1	0.9–1.2
High salt intake						
No	1		1		1	
Yes	0.6	0.5–0.8	0.6	0.4–0.8	0.7	0.5–0.9
Abusive consumption of alcohol						
No	1		1		1	
Yes	2.5	1.9–3.4	2.3	1.6–3.3	2.4	1.4–4.3
Hypertension						
No	1		1		1	
Yes	6.2	5.5–6.9	6.1	5–7.4	6.1	5.3–7.1
High cholesterol						
No	1		1		1	
Yes	4.1	3.7–4.6	4	3.3–4.9	4.1	3.6–4.7
Assessment of the state of health						
Good	1		1		1	
Regular	4.8	4.2–5.4	5	4.1–6.1	4.5	3.9–5.3
Poor/very poor	7.4	6.4–8.7	5.9	4.5–7.8	8.1	6.7–9.7

BMI: body mass index.

^a^ The categories yellow and indigenous were excluded from these analyses.

^b^ 32% missing data for BMI.

^c^ Physical activity domains: leisure, work, commute to work, and home.

To consider possible factors in the interpretation of these associations, adjustments were made for differences of age, sex, and schooling, as presented in Table 3. The increase by age group was higher in men than in women. Associations with factors that increase progressively with age, such as hypertension, high cholesterol and self-perception of poor health were lower after the adjustments, but remained statistically significant. Differences regarding eating habits and physical activity indicators lost significance, but overweight, obesity, alcohol consumption and smoking cessation remained associated. The inverse association between schooling and self-reported diabetes, observed in women, remained the same after these adjustments.

**Table 3:** Adjusted prevalence ratiod and 95% confidence interval for diabetes in adults stratified by sex, according to sociodemographic characteristics, clinical conditions, and lifestyles. National Health Survey, Brazil, 2013.

Variable	Total		Male		Female	
		
	adjPR^d^	95%CI	adjPR^e^	95%CI	adjPR^e^	95%CI
Age (years)						
18–24	1			1	1	
25–34	1.7	1–2.9	2	0.9–4.8	1.4	0.7–2.8
35–44	5.8	3.4–9.7	6.9	3.2–14.9	5	2.5–10.1
45–54	12.6	7.8–20.4	15.9	7.7–33.2	10.3	5.3–20
55–64	25.8	15.7–42.5	35.1	16.8–73.3	20.1	10.3–39.3
≥ 65	37	22.6–60.6	56.4	27.2–116.9	26.4	13.5–51.7
Schooling (years)						
Illiterate/some elementary or middle school	1				1	
Elementary or middle school/some high school	1.2	1–1.4	1.7	1.3–2.4	0.9	0.7–1.1
High school/some college	0.9	0.7–1	1.4	1.1–1.8	0.6	0.5–0.8
College degree	0.7	0.6–0.9	1.3	0.9–1.8	0.4	0.3–0.6
Race/color^a^						
White	1				1	
Black	1.1	0.9–1.4	1.1	0.7–1.6	1.1	0.9–1.4
Pardo	1	0.3–1.1	0.9	0.8–1.1	1	0.8–1.1
Categories of BMI^b^						
Underweight/normal (< 25 kg/m^2^)	1				1	
Overweight (between 25 and 29.9 kg/m^2^)	1.8	1.5–2.2	1.7	1.3–2.2	1.9	1.5–2.3
Obesity (≥ 30 kg/m^2^)	2.9	2.4–3.5	2.7	2.1–3.6	2.9	2.4–3.7
Insufficiently active in 4 households^c^						
No	1				1	
Yes	1	0.9–1.1	1.1	0.9–1.4	0.9	0.8–1
Smoking						
Non-smoker	1				1	
Ex-smoker	1.3	1.2–1.5	1.3	1–1.6	1.4	1.2–1.6
Smoker	0.9	0.7–1.1	0.9	0.7–1.3	0.8	0.7–1
Recommended consumption of fruits and vegetables (5 or more servings per day)						
Yes	1				1	
No	0.9	0.8–1	0.9	0.7–1.1	0.9	0.7–1
Consumption of red meat with fat						
Yes	1				1	
No	1.1	1–1.3	1.2	1–1.5	1	0.9–1.2
High salt intake						
No	1				1	
Yes	0.9	0.8–1.1	0.8	0.6–1.1	1	0.8–1.3
Abusive consumption of alcohol						
No	1				1	
Yes	1.5	1.1–2	1.5	1.1–2.2	1.3	0.7–2.2
Hypertension						
No	1				1	
Yes	3.1	2.8–3.6	3.1	2.5–3.8	3.1	2.6–3.6
High cholesterol						
No	1				1	
Yes	2.6	2.3–2.9	2.7	2.2–3.3	2.4	2.1–2.8
Assessment of the state of health						
Good	1				1	
Regular	3.2	2.7–3.6	3.5	2.8–4.4	2.9	2.5–3.4
Bad/very bad	4	3.4–4.9	3.6	2.6–5.1	4.2	3.3–5.2

BMI: body mass index.

^a^ Excluded the categories yellow and Indian.

^b^ 32% missing data for BMI.

^c^ Physical activity domains: leisure, work, commute to work, and home.

^d^ PR adjusted by age, sex, and schooling.

^e^ PR adjusted by age and schooling.

## DISCUSSION

Data of the PNS, held in 2013, estimate that 6.2% of individuals with 18 years or more have received medical diagnosis of diabetes, which suggests that 9.1 million Brazilian adults have diabetes^b^.

Type 2 diabetes is the result of an interaction between genetic and environmental factors^9^. Among the factors of context, we highlight the rapid transformations in eating patterns, with the introduction of foods rich in fat and simple carbohydrates, as well as a reduction in physical activity levels, resulting in the accelerated increase of overweight and obesity^10,11^. To stop diabetes, measures aiming at behavioral changes are fundamental, such as increased intake of coffee, integral grains, fruits and nuts; reduction of saturated and trans fat intake, refined grains, red or processed meat, and sugary drinks; moderate consumption of alcohol; implementation of systematic physical activity; weight maintenance; and reduction of the habit of smoking^4,12^.

Among the factors associated with diabetes, this study identified a greater prevalence among women. This finding is common in self-report studies as the increased use of health services by women results in a greater opportunity for medical diagnosis, and has been reported in other Brazilian studies^1,13^. However, a greater prevalence in women is not uniformly reported in the literature, especially when biochemical measurements and adjustments for other sociodemographic characteristics are used^14,15^. In the Brazilian Longitudinal Study of Adult Health (ELSA-Brasil), conducted in six Brazilian cities and using biochemical measures, Schmidt et al. found a 43% higher prevalence of diabetes among men^16^.

The positive association between diabetes and increasing age is already considerably based on the literature^4,9,10,17,c^. In the age group above 65 years, diabetes is known to be present in about a fifth of the population, a fact that can be justified by the changes inherent in the aging process, by the reduction of physical activity and lifelong unhealthy eating habits. Additionally, this age group has a greater opportunity for diagnosis, especially in men, due to screening of the disease, which is indicated for everyone after 45 years of age^2,d^.

On the other hand, the prevalence of diabetes, as well as that of the metabolic syndrome, associated with cardiovascular diseases in adult life has also been rising in younger populations^18-20^. The increase in the prevalence of obesity in adolescence in recent years explains, to a large extent, the progress of type 2 diabetes mellitus in young populations.

The inverse relation between self-reported diabetes and schooling has been described in other Brazilian studies^1,13,14,e^. The study by Schmidt et al.^16^ also showed inverse relation even when considering the total prevalence of diabetes. Similar results were found in international studies, such as the Alameda County Study, published in 2005 by Maty et al.^21^, which indicated an association between schooling and diabetes mellitus, after adjustment for income and occupation variables. Higher schooling can be a protective factor for diabetes due to the increased access to health promotion practices, such as healthy eating habits and physical activity, in addition to greater access to health services^9,22^.

The crude prevalence of diabetes was lower in *pardos*, but when adjusted by age, schooling and sex, the differences according to race/color were not significant. This relation is inconclusive in the literature. Although some studies indicate higher prevalence of diabetes among black adults^14,16^, recent analyses of Vigitel (telephone survey) also did not find association of diabetes according to race/color^23^. Again, differences in disease detection methods can influence this result. In ELSA-Brasil, the prevalence of diabetes, determined by report and also by laboratory tests, was 9% higher in *pardos* and 38% higher in black people than in white people^16^. The consumption of 400 g per day of fruits and vegetables is recommended by the WHO to reduce the incidence of cardiovascular diseases and certain types of cancer and also to prevent and treat overweight and diabetes^a^. These foods provide the least amount of saturated fat and cholesterol, being appropriate in the diabetic diet^4^, as opposed to the consumption of fatty foods, which constitute a risk factor for cardiovascular diseases and obesity^4,a^. In this study, the association between aspects of food consumption and diabetes disappeared after adjustment for age, schooling and sex, suggesting that eating patterns were differential according to these characteristics, rather than directly associated with diabetes.

A sedentary lifestyle has been associated with insulin resistance in non-diabetic individuals, regardless of obesity^24^. The regular practice of physical activity is important for the treatment and reduction of diabetes^4^, as it increases the number of capillaries and muscle fibers, facilitating glucose transport and uptake^24^. In this study, after the adjustments, the indicator of physical inactivity lost significance, a finding possibly explained by reverse causality, considering encouragement to perform regular physical activity in this population^2^.

The association between adiposity, especially visceral, and diabetes is well documented^4,22^. However, studies also indicate association between diabetes and overall obesity as characterized by high body mass index^9^. The pathophysiological mechanisms that result in the association between obesity and diabetes are complex and multifactorial. Among them is the infiltration of fat in the liver, interfering in the hepatic metabolism and increasing insulin resistance. In the pancreas, excess of circulating fats and glucose results in increased demand for insulin secretion, leading to stress and eventual exhaustion of the β-cells, insulin producers, which worsens the situation^4^.

The association of diabetes and obesity suggests a lifestyle among Brazilian adults that is now common in urbanized western societies, since the excess of weight reaches more of the half of the Brazilian adult population, and obesity about 17.4%^25^. The study identified a gradient of association with increasing adiposity, the PR being about twice that of normoweight individuals among overweight people and three times among obese people.

Tobacco is known to be a strong risk factor for cardiovascular diseases^a^. The amount and duration of smoking correlate directly with the progression of diabetes and cardiovascular complications, and smoking cessation is a fundamental and priority measure in secondary prevention^4,26^. In this study, we found an association between diabetes and ex-smokers. This could be explained in two ways: first, people with the diagnosis of diabetes stop smoking due to the illness; second, the smoking would increase the risk of developing diabetes, but the disease would manifest especially after the weight gain associated with the smoking cessation^27^.

The association between abusive consumption of alcohol and diabetes can occur by the direct action of alcohol^8^ or confounding by other unhealthy living habits present in individuals with high alcohol intake. In this study, individuals who reported diabetes, especially men, were the ones that most reported abusive consumption of alcoholic beverages. Thus, the health team should consider this factor in the strategies of glycemic control of these patients.

Hypertension is recognized as an important risk factor for diabetes and may contribute to both micro and macrovascular lesions^4^. The question concerning hyperlipidemia in the PNS refers to high cholesterol or triglycerides, without distinction between both or among the fractions of cholesterol. High total cholesterol and LDL cholesterol are not generally considered diabetes risk factors, while low levels of HDL-C and high triglyceride are^9^. The association with high cholesterol or triglycerides shown here can represent reverse causality, arising from the fact that the investigation of dyslipidemia is more frequent in carriers of other risk factors for cardiovascular disease, such as diabetes.

The occurrence of chronic diseases in general has been related to the assessment of a “poor” state of health^b,e^. This is a qualitative and synthetic health indicator, but with a good ability to predict more serious outcomes^7^. A previous Brazilian study^7^ indicated diabetes prevalence up to four times higher in adults with a self-perception of poor health, results very close to the ones found in this study. This relation can be associated with both the presence of characteristic symptoms of the disease and its complications and with the changes implemented because of the disease, such as the high number of visits to doctors and health services, lifestyle changes, medications and, often, limitations in daily activities, leading to a greater awareness of the worsening of the state of health^7^.

Due to high morbidity, diabetes goes along with reduced quality of life and incapacities, and it is estimated that the disease results in 89 million quality-adjusted years of life lost in the world. Morbidity results predominantly from macrovascular complications – ischemic heart disease, cerebrovascular accident and peripheral artery disease – and microvascular ones, including retinopathy, nephropathy, and neuropathy, which can lead to vision loss, kidney failure and chronic pain, respectively^28-30^.

Cardiovascular disease is the leading cause of morbidity and mortality in diabetic patients^31,32^. The prevention of these diseases involves, among other things, the treatment of other cardiometabolic risk factors associated with diabetes, such as hypertension, dyslipidemia, obesity, smoking, and sedentariness^2,4^.

Some characteristic limitations of cross-sectional studies can be highlighted in relation to this study’s results. The reverse causality bias, caused by simultaneous measurements of risk or protection factors and of outcomes, limited inferences about the directionality of causality. The use of self-reported morbidity data depends on the access to health services for the diagnosis; thus, users who more often use these services have a higher opportunity for medical diagnosis of diabetes.

Despite the limitations of cross-sectional studies, the results of the PNS detailed in this study, allow the establishment of a set of factors associated with diabetes, contributing to the rational construction of public policies for prevention and health promotion. After adjustment by age, schooling and sex, diabetes was shown to be associated with increasing age, lower schooling, overweight and obesity, and hypertension, indicating a pattern of common risk factors to the occurrence of other chronic diseases in the adult Brazilian population. The close association between diabetes and assessment of the state of health as poor shows the importance of diabetes in the quality of life of Brazilian adults and older adults. This aspect, in addition to the forecast of an increase in the prevalence of the disease in the medium term, is worrying.

The information of PNS, by being representative of the Brazilian population, will be useful to support the formulation of public policies in the areas of promotion, vigilance and attention to health of the Brazilian Unified Health System (SUS), aligned with the Strategic Action Plan to Combat Non-Communicable Diseases, in Brazil, in the period 2011-2022^f^ and the Global Plan to Combat Chronic Non-Communicable Diseases of the WHO in 2013^g^, setting targets to reduce the burden of these diseases^a^.

With the rapid aging of the Brazilian population, we reinforce the importance of early intervention to prevent and control diabetes, along with the investment in health promotion programs, such as interventions for healthy eating, restriction of tobacco and alcohol consumption, and physical activity practice programs, which will help to reduce overweight and obesity and other risk factors common to chronic non-communicable diseases in general.

## Footnotes

a World Health Organization. Global status report on noncommunicable diseases 2014. Geneva: WHO; 2014 [cited 2015 Jan 29]. Available from: http://www.who.int/nmh/publications/ncd-status-report-2014/en/

b Instituto Brasileiro de Geografia e Estatística. Pesquisa Nacional de Saúde 2013: percepção do estado de saúde, estilos de vida e doenças crônicas. Brasil, grandes regiões e unidades da federação. Rio de Janeiro: IBGE; 2014 [cited 2015 Jan 9]. Available from: http://www.ibge.gov.br/home/estatistica/populacao/pns/2013/

c Ministério da Saúde (BR), Secretaria de Vigilância em Saúde, Departamento de Doenças e Agravos não Transmissíveis e Promoção da Saúde. Vigitel Brasil 2014: vigilância de fatores de risco e proteção para doenças crônicas por inquérito telefônico. Brasília (DF): Ministério da Saúde; 2015 [cited 2015 Jan 9]. Available from: http://bvsms.saude.gov.br/bvs/publicacoes/vigitel_brasil_2014.pdf

d Ministério da Saúde (BR), Secretaria de Atenção à Saúde, Departamento de Atenção Básica. Estratégias para o cuidado da pessoa com doença crônica: diabetes mellitus. Brasília (DF): Ministério da Saúde; 2013 [cited 2015 Jan 21]. (Cadernos de Atenção Básica, 36). Available from: http://bvsms.saude.gov.br/bvs/publicacoes/estrategias_cuidado_pessoa_diabetes_mellitus_cab36.pdf

e Instituto Brasileiro de Geografia e Estatística. Pesquisa Nacional por Amostra de Domicílios. Um panorama da saúde no Brasil: acesso e utilização dos serviços, condições de saúde e fatores de risco e proteção à saúde: 2008. Rio de Janeiro: IBGE; 2010 [cited 2015 Jan 9]. Available from: http://bvsms.saude.gov.br/bvs/publicacoes/pnad_panorama_saude_brasil.pdf

f Ministério da Saúde (BR), Secretaria de Vigilância em Saúde, Departamento de Análise de Situação de Saúde. Plano de ações estratégicas para o enfrentamento das doenças crônicas não transmissíveis (DCNT) no Brasil 2011-2022. Brasília (DF): Ministério da Saúde; 2011 [cited 2012 Jan 11]. (Série B. Textos Básicos de Saúde). Available from: http://bvsms.saude.gov.br/bvs/publicacoes/plano_acoes_enfrent_dcnt_2011.pdf

g World Health Organization. Global action plan for the prevention and control of NCDs 2013-2020. 10. ed. Geneva: WHO; 2013 [cited 2014 Jan 27]. Available from: http://www.who.int/nmh/events/ncd_action_plan/en/
